# Harnessing Dendritic Cells for Poly (D,L-lactide-*co*-glycolide) Microspheres (PLGA MS)—Mediated Anti-tumor Therapy

**DOI:** 10.3389/fimmu.2019.00707

**Published:** 2019-04-05

**Authors:** Julia Koerner, Dennis Horvath, Marcus Groettrup

**Affiliations:** ^1^Division of Immunology, Department of Biology, University of Konstanz, Konstanz, Germany; ^2^Biotechnology Institute Thurgau at the University of Konstanz, Kreuzlingen, Switzerland

**Keywords:** PLGA, microspheres, cancer vaccine, dendritic cell, anti-tumor response, spray drying, immunotherapy, CTL

## Abstract

With emerging success in fighting off cancer, chronic infections, and autoimmune diseases, immunotherapy has become a promising therapeutic approach compared to conventional therapies such as surgery, chemotherapy, radiation therapy, or immunosuppressive medication. Despite the advancement of monoclonal antibody therapy against immune checkpoints, the development of safe and efficient cancer vaccine formulations still remains a pressing medical need. Anti-tumor immunotherapy requires the induction of antigen-specific CD8+ cytotoxic T lymphocyte (CTL) responses which recognize and specifically destroy tumor cells. Due to the crucial role of dendritic cells (DCs) in initiating anti-tumor immunity, targeting tumor antigens to DCs has become auspicious in modern vaccine research. Over the last two decades, micron- or nanometer-sized particulate delivery systems encapsulating tumor antigens and immunostimulatory molecules into biodegradable polymers have shown great promise for the induction of potent, specific and long-lasting anti-tumor responses *in vivo*. Enhanced vaccine efficiency of the polymeric micro/nanoparticles has been attributed to controlled and continuous release of encapsulated antigens, efficient targeting of antigen presenting cells (APCs) such as DCs and subsequent induction of CTL immunity. Poly (D, L-lactide-*co*-glycolide) (PLGA), as one of these polymers, has been extensively studied for the design and development of particulate antigen delivery systems in cancer therapy. This review provides an overview of the current state of research on the application of PLGA microspheres (PLGA MS) as anti-tumor cancer vaccines in activating and potentiating immune responses attempting to highlight their potential in the development of cancer therapeutics.

## Introduction

With an annual incidence of several million new cases worldwide, cancer represents one of the most prevalent malignancies and leading causes of pain and mortality. Conventional treatment options usually include a combination of primary resection, radiotherapy and/or chemotherapy. However, cancer patients suffer from devastating adverse side-effects and poor quality of life after chemo- or radiation therapy. Moreover, therapeutic failure of standard therapeutics results in increased risk of tumor relapse and metastasis formation (1). Hence, there is an urgent need for safe and effective vaccine development against this life-threating group of diseases. With the identification of multiple unique cancer antigens (tumor-associated antigens, TAA) and the investigation of manifold immune evasion pathways of tumors, immunotherapy has become a growing focus in clinical research.

Cancer immunotherapy encompasses therapeutic modulation of the host's immune system to defend against foreign or self-antigens that have gone awry in tumor development. Cancer vaccines aim at triggering immune activation to specifically target and eliminate tumor cells. Ideally, a memory response is generated to impede metastasis formation and further spread of the disease. In contrast to passive immunotherapy which aims at delivering neutralizing antibodies, active forms of immunotherapy are supposed to induce multi-faceted cell mediated immunity by simultaneous activation of APCs, CD4+ and CD8+ T cells, as well as B cells and innate immune cells, as for instance NK cells, granulocytes and macrophages. Compared to standard tumor therapies, immunotherapeutic anti-tumor vaccines offer distinct advantages, namely: increased specificity and reduced toxicity by activation of antigen-specific CTL responses. Effector CTLs are able to decrease large tumor masses and induce long-term protection against tumor recurrence through induction of immunological memory (2). Recent advances in cancer immunotherapy have paved the way for the discovery of versatile methods for prevention or treatment of various types of cancer. As a result, several cancer vaccines are currently investigated in clinical trials. However, most of them have not progressed beyond phase III studies. Although antigen-specific responses were generated and increases in overall survival rates were obtained, there is no consistency in clinical benefit. Most of the approaches were presented with major drawbacks in vaccine delivery and efficacy. Administration of soluble antigenic formulations, e.g. synthetic peptides or purified tumor-associated antigens was not promising due to poor immunogenicity, limited bioavailability, short half-life and rapid degradation or elimination of the antigens *in vivo*, demanding the need for repeated injections (3).

Due to the unique ability of DCs to prime and activate naïve T cells (4, 5), DC-based vaccination strategies have shown to be a promising approach in the development of polyvalent cancer vaccines. The first promising results have been achieved using *ex vivo* derived autologous tumor cells or DCs that have been pulsed with various tumor-associated proteins or peptides (6). However, major drawbacks were seen in suboptimal antigen presenting capacity of isolated DCs or simple lack of autologous tumor samples (7, 8). Several promising immunotherapeutic advances came across with the use of allogeneic tumor-lysate pulsed DCs, loading of DCs with MHC class I restricted tumor antigens (9–11), or via transfection of cDNA encoding TAAs (8, 12). Whole tumor lysate contains a large repertoire of tumor antigens capable of inducing immune responses against a broad spectrum of multiple epitopes including those that are unique to the patient's tumor. The development of DC-based vaccination has led to the first therapeutic cancer vaccine. In April 2010, Provenge® (Sipuleucel-T) was approved by the FDA for treatment of castration-resistant, metastatic prostate cancer (13). This immunotherapy involves *ex vivo* stimulation of autologous, blood-derived antigen presenting cells from prostate cancer patients that are pulsed with a prostate cancer-associated antigen [PAP (prostate acid phosphatase)–GM-CSF fusion protein]. DCs were subsequently re-introduced into patients to stimulate an immune response against PAP-expressing prostate cancer cells. These well-tolerated approaches using *ex vivo* loaded DCs were tested in a variety of experimental models and clinical trials [reviewed in Tacken et al. (14)], and seemed to be encouraging due to good safety records, the generation of enhanced T cell responses and partial reduction of tumor load. However, clinical application is still limited as these *ex vivo* procedures are laborious and time-consuming, extremely expensive and lack universal applicability (15). More importantly, the overall clinical response rates in cancer patients were only 7% (16).

To circumvent the limitations associated with *in vitro* manipulation of cells, direct *in vivo* targeting of DCs along with appropriate adjuvants for simultaneous activation of dendritic cells has gained major focus. Particulate delivery systems have shown to overcome the main obstacles related to traditional cancer therapeutics. Instead of causing the risk to induce systemic, adverse immunity, vaccine antigens are delivered to DCs in a targeted manner. We and others have established the use of PLGA MS as an efficient vaccine delivery system for dendritic cell targeting. Subsequent induction of potent immune responses has led to remarkable protective and therapeutic anti-tumor activity *in vivo*. In this article we review how DCs can be antigen charged and matured with PLGA MS *in vitro* and *in vivo* and how microspheres can be produced and formulated to optimally be taken up by DCs. Moreover, we discuss the parameters how antigen presentation and T cell stimulation by PLGA MS-loaded DCs can be improved to elicit a vigorous and effective anti-tumor immune response.

## Comparison of Particulate Antigen Delivery Systems

At present, several particulate drug delivery systems for cancer immunotherapy–other than PLGA based particles–have passed pre-clinical investigations and are currently tested for human application, such as liposomes, virosomes, immune-stimulatory complexes (ISCOMs) or gold particles. These systems are reviewed elsewhere (17) and are beyond the scope of this article. Furthermore, detailed analysis of nano-sized particulate vaccine delivery systems has been already extensively reviewed (18–20) and is only of specialized focus in this review.

Multiple different natural or synthetic polyesters have been reported for the development of (sub)micron sized colloidal drug delivery systems, such as poly(lactic acid) (PLA), poly(glycolic acid) (PGA), poly(ε-caprolactone) (PCL), poly(methyl methacrylate) (PMMA), poly(β-amino esters) as well as other ester derivates [poly(anhydrides), poly(orthoesters), poly(phosphoesters), poly(phosphazenes) or poly(cyanoacrylate)]. Due to their excellent bioavailability, biodegradable and biocompatible properties, controlled release and low toxicity, these polymers have been extensively studied as delivery systems of various therapeutic vaccines as well as for cancer immunotherapy in preclinical settings (21–23). Based on the method of preparation, different types of polymeric particles can be designed: spheres, capsules, cubes and other shapes. While the active compound of micro/nanocapsules is contained inside a cavity underneath the polymeric layer, micro/nanospheres homogenously entrap the encapsulated materials into the inner polymer matrix core (24).

**Figure 1:**
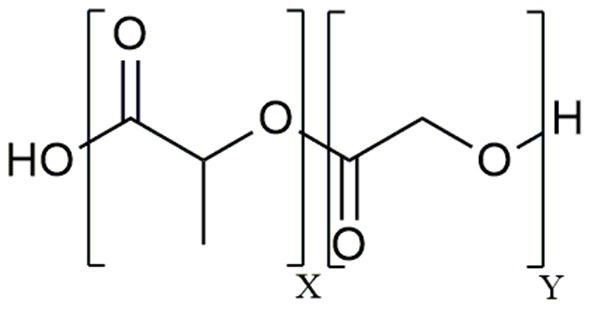
Chemical structure of the poly (D,L – lactide-*co*-glycolide) (PLGA) co-polymer.

The aliphatic co-polymer PLGA is one of the most frequently used and explored polymers for controlled delivery of bioactive molecules in microspheres and nanoparticles (NP) (25). The amorphous PLGA is composed of varying proportions of lactic and glycolic acids (Figure 1). Due to its ideal *in vivo* properties of biodegradability, biocompatibility and its clear safety records, PLGA has been licensed by the U.S. Food and Drug Administration (FDA) and European Medicines Agency (EMA) for use in pharmaceutical application via parenteral (subcutaneous, intradermal, intramuscular) and mucosal routes as well as for suspension formulations of biomedical devices including surgical sutures and bone implants (26). At present, there are 12 PLGA-based microparticle cancer vaccine formulations approved by the FDA for clinical use. Most of these PLGA MS systems are targeting prostate cancer, for example Pamorelin LA® which encapsulates the gonadotropin releasing hormone (GnRH) agonist triptorelin pamoate for palliative treatment of advanced prostate cancer (27). Of note, not a single nanoparticulate vehicle has reached clinical approval due to associated toxicity issues (28) as discussed later in this review.

## Properties of PLGA Particles

A wide range of biologically active compounds including hormones, antibiotics, and drugs can be encapsulated into PLGA particles (29, 30). Thus, PLGA micro- and nanoparticles have been well-established as delivery systems of innumerable antigens such as proteins, peptides, lipopeptides, viral or bacterial DNA as well as immunomodulatory molecules (31–37). PLGA particles exhibit a vast array of advantages over soluble vaccine formulations. At first, GMP (good manufacturing practice) grade polymer is commercially available (for example, PLGA Resomer® from Evonik Industries) meeting the GMP requirements of regulatory authorities. Encapsulation within PLGA particles protects the encapsulated bioactive molecules from premature degradation by proteolytic enzymes or metabolic turnover and minimizes loss of therapeutic activity prior to delivery. The enhanced bioavailability is due to sustained and controlled release of encapsulated substances over extended time periods of several weeks to months thus creating a depot effect at the site of injection. Prolonged antigen presentation and continuous T cell stimulation would circumvent the need for conventional multiple dose immunization schedules, e.g. prime-boost vaccination (38, 39). Hence, PLGA MS would provide a valuable approach for single administration vaccine design so that clinical intervention is only limited to one therapeutic injection. Encapsulation of peptides into PLGA MS was shown to enhance and extend antigen presentation on MHC class I and II by DCs and macrophages (29, 38) which is possibly due to higher total load of antigens and prolonged degradation time of larger microparticles compared to nanoparticles (40). Furthermore, entrapment of proteins or peptides into biodegradable PLGA microspheres increases the immunogenicity of poorly immunogenic antigens, e.g. weak self-antigens in tumor tissue. While soluble peptide immunizations elicited only very poor CD8+ T cell responses, microencapsulation of the HLA-A^*^0201 restricted immunodominant epitope STEAP 1 (six transmembrane epithelial antigen of the prostate) was shown to induce potent prostate cancer peptide-specific CTL activation and cytotoxic effector function (36, 41).

### Release and Encapsulation Qualities of PLGA Microspheres

Upon encountering aqueous medium, PLGA is slowly hydrolyzed into its original monomeric components. The resulting products lactic and glycolic acid are physiological metabolites of the citric acid cycle and thus completely eliminated from the human body as carbon dioxide and water (42). The degradation rate and subsequent drug release is primarily dependent on the PLGA polymer composition and the molecular weight of the polymer. These two factors also impact hydrophilicity, the hydration rate as well as the glass transition temperature (Tg) of the respective polymer type, which in turn also affect the release profile (43). A high content of glycolic acid in the co-polymer leads to higher hydrolysis rates and a more rapid release, as glycolic acid is slightly more hydrophilic than the crystalline lactic acid, which fosters water permeability into the polymer matrix. Several other factors contribute to release rates of PLGA MS including concentration of the polymer in the organic solvent during PLGA MS fabrication, PLGA particle size and morphology, as well as storage conditions such as temperature and humidity and of course, the encapsulated material itself. The PLGA 50:50 polymer is preferred over other PLGA polymers with different lactic:glycolic ratios (65:35; 75:25; 80:20) in controlled release vaccine formulation since encapsulated molecules are homogenously dispersed inside the polymer matrix. Additionally, it is slightly more hydrophilic and thus possesses the fastest degradation rate resulting in complete degradation within 30 to 60 days in aqueous medium (44). It also occupies the least crystallinity hence being more prone to (enzyme-independent) hydrolysis and bulk erosion. Only when the PLGA polymer becomes porous and hydrated, encapsulated material of high molecular weight can be released. This will prevent early release of antigens or adjuvants before internalization of the particles by DCs and thus reduces systemic distribution of the encapsulated molecules. The release profile of PLGA degradation encompasses two phases with an initial burst that is followed by progressive release of the encapsulated material. The burst release is likely attributed to weakly bound or adsorbed proteins on the PLGA particle surface that are rapidly dispersed upon submersion into aqueous media (45). Noticeably, about 30% of the entrapped material can be released within a few days, though the percentage markedly depends on the physical properties of the microparticles (46).

### Physico-Chemical Characteristics of PLGA Particles

A major advantage of using PLGA polymers is attributed to its great flexibility and ease to manipulate and modify its physicochemical properties such as: molecular mass of the polymer, hydrophilicity and crystallinity (monomer ratio), end-group chemistry, particle size and surface charge. All these factors can be modified to obtain desired and suitable degradation rates and subsequent release patterns for individual treatment regimen. Furthermore, these properties also dictate intracellular trafficking and can thus be individually adjusted to the needs of the encapsulated material (47). The main improvement of using PLGA particles as vaccine delivery system relies on the ability to simultaneously stimulate innate and adaptive immunity through directing intracellular antigen processing toward the cross-presentation pathway. Furthermore, maintenance of integrity and thus activity of the encapsulated material ensures their bioavailability and their ability to mount effective immune responses (48).

## Production Methods for PLGA MS

There are several methods employed to produce micro- and nanoparticles such as emulsification-solvent-evaporation, organic phase separation (coacervation), nano-precipitation (diafiltration), and newer strategies such as supercritical microfluidics, coaxial electrospray or the PRINT (particle replication in non-wetting templates) technology (49). However, major drawbacks of the most widely used single or double emulsification solvent evaporation techniques is poor encapsulation efficiency, which either requires increased drug loading or the use of surfactants (e.g. PVA, poly-vinyl alcohol) to stabilize the oil-in-water emulsion until particles have been formed. Moreover, high shear or cavitation forces, excessive use of energy or freezing and drying cycles cause significant risk of aggregation or degradation of encapsulated material of these particles, thereby rendering emulsion techniques difficult for mass production (50). Furthermore, the initial burst is very high due to poor drug loading into the particles while adsorption onto the particle surface is very common (22). Nevertheless, we have used and optimized the spray-drying technology in our laboratory.

### Microencapsulation by Spray Drying

**Figure 2:**
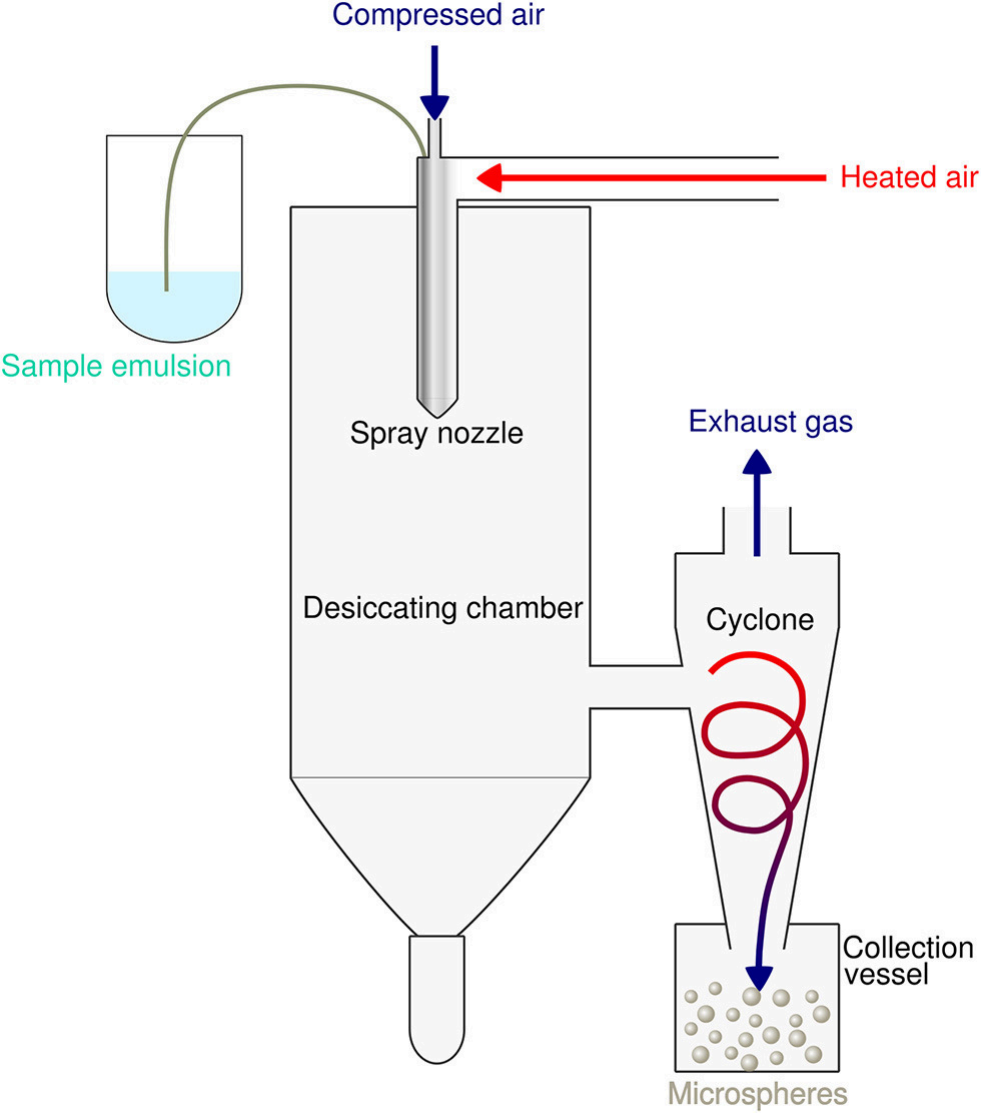
Schematic illustration of the spray drying process for preparation of PLGA microspheres. The sample emulsion is spray-atomized into small droplets at the nozzle. These droplets in the heated dry air flow are transformed into dry particles by evaporation of the organic solvent. The particles are then separated from the drying medium and collected under constant low pressure as dry powder in the lower collection vessel.

Spray-drying is a very suitable and rapid one-step process for encapsulation of both hydrophobic as well as hydrophilic proteins and peptides into PLGA particles. The principle is based on nebulization of a solid-in-oil dispersion or water-in-oil-emulsion composed of antigen and adjuvants in an aqueous phase that is mixed with the volatile, water-immiscible organic solvent [e.g. dichloromethane (DCM)] used to dissolve the PLGA polymer. The fluid is spray-atomized into a gas stream of compressed air or compressed nitrogen into a desiccating chamber, where liquid droplets pass a current of warm air-stream subsequently creating microparticles at the spray nozzle by evaporation of the organic solvent (51, 52). Evaporation keeps the product temperature at low levels, thus only little temperature deterioration occurs (53). As the fluid is converted into a dry powder in the drying chamber, the particle-loaded air stream enters tangentially into the cyclone, which results in a centrifugal force that creates a downward spiral movement in the cyclone causing particle deposition at the bottom of the cyclone separator and the collecting vessel (54) (see Figure 2).

This microencapsulation process warrants stability and the biological activity of the encapsulated epitopes and guarantees high yield and encapsulation efficiencies of more than 85% (55). The low preparation temperature of the spray drying method avoids thermal denaturation of encapsulated compounds. The produced microspheres do not exhibit aggregation and show good suspensibility in injection solution. Spray drying usually produces particles with a particle size distribution of about 500 nm−5 μm. Besides process parameters such as the liquid feed rate, the drying air flow rate or the inlet air temperature, molecular weight and concentration of the polymer in the organic solvent critically determines particle size and affects microsphere morphology and subsequently degradation and drug release of PLGA MS (56). The spray-drying method has been successfully used with several biodegradable polymers such as PLA, PCL, gelatin, and polysaccharides or related biopolymers (57–59). It has several advantages over other particle production methods such as lower residual organic solvent, higher yield, and higher encapsulation efficiency or prolonged sustained release. Mentionable, particle sizes can now be easily controlled by using the nano-spray drying method based on the vibrating mesh technology (60, 61). Sticky adhesion of PLGA microparticles to the interior surface of the spray-drier's glass ware, as always referred to a salient drawback of spray drying (62), has been overcome by the use of the non-ionic surfactant Poloxamer® 188 to wash out spray-dried particles.

The optimized drying procedure after spray drying by vacuum drying over several days reduces the residual amount of organic solvent to a minimum (63). The authorized pharmaceutical limit for residual organic volatile impurities of DCM by the U.S. (USP) and European Pharmacopeia (PhEur) is 0.06%. This is pertinent, as incomplete solvent removal or solvent impurities may cause chemical degradation of the encapsulated compounds within the polymer matrix. By efficient removal of the solvent, spray-dried PLGA MS are highly stable as dry powder for long-term storage without degradation of the encapsulated compounds thus preserving therapeutic activity. Furthermore, spray drying can be easily scaled up to produce large batches. Polymer-drug solutions of high volumes are rapidly spray-dried within minutes, which would facilitate industrial production processes for potential clinical application.

### *In vivo* Uptake of PLGA Particles by APCs

Without specific recognition, PLGA MS provide non-specific and untargeted antigen delivery toward APCs (mainly DCs, but also macrophages) because particle sizes of 0.5–5 μm exhibit similar dimensions to pathogens (44, 64). Conceptionally, the particulate matter facilitates cellular uptake and internalization by APCs and allows for faster degradation and rapid intracellular release of the antigenic cargo (25). Thus, encapsulated antigens are better processed and presented by APCs compared to antigens in soluble form. Consequently, PLGA MS-mediated antigen delivery induces a more efficient recognition of presented epitopes by the immune systems (65, 66). DCs, but also macrophages, are highly phagocytic cells being equally able to internalize large, micron-sized particles and small nanoparticles. Several studies indicate that the majority of DCs are able to take up PLGA MS (as well as PLGA nanoparticles) within 24 h. Although, the ideal particle size for uptake by APCs still remains a matter of debate, the particle size critically influences cellular uptake mechanisms but also dictates fate of intracellular endocytic pathways and DC activation and thus affects the generated immune response (20).

### Particle Size Influences the Immunogenicity of PLGA Particles

It has been demonstrated that DCs preferably engulf smaller, submicron- or virus–sized particles of 20–200 nm, whereas large particulate vaccines of bacterial size (>500 nm; e.g. microspheres) are mainly taken up by macrophages (67, 68). PLGA particle uptake by human DCs *in vitro* was less efficient at sizes exceeding 500 nm (69). A comparative study by Joshi et al. analyzed OVA (ovalbumin) -specific CTLs in blood after *in vivo* administration of PLGA particles containing OVA/CpG of 300 nm, 1, 7, and 17 μm size. The smallest particles induced the highest antigen specific T cell response suggesting that the smaller the particle the stronger the response (70). Noteworthy, PLGA particles were injected intraperitoneally and tetramer-positive signals were analyzed after two booster vaccinations–incomparable to our vaccination regimen and analysis of peak T cell response on day 6 after PLGA MS vaccination *in vivo* (71). In fact, it was reported, that immature DCs (iDCs) are also able to internalize larger particles by either phagocytosis or micropinocytosis (72, 73). As well, Gutierro et al. have demonstrated increased access of large sized PLGA particles (1 μm) to APCs which in turn elicited a higher total IgG response and increased IFN-γ production of T cells (74). We and others have demonstrated efficient uptake of PLGA microparticles by human peripheral blood monocyte-derived DCs (moDCs), murine immature bone-marrow derived DCs (BMDCs), as well as macrophages *in vitro* and by CD11c+ dendritic cells after subcutaneous immunization *in vivo* (75–77). The entrapped content in DCs is efficiently transported from peripheral tissue to the site of antigen-presentation in secondary lymphoid organs (SLOs) like spleen and liver, providing direct evidence for migration of immature, skin-resident DCs to draining lymph-nodes after PLGA MS uptake (78). This was experimentally confirmed by the presence of Quantum-Dot (QD) positive PLGA microspheres in CD169+ subcapsular sinus macrophages (SSM) in draining lymph nodes (dLN) after immunization with these fluorescent microspheres (79). In contrast to subcutaneous PLGA administration into dermis or epidermis, macrophages are the predominant cell type entrapping PLGA particles after i.p. administration (80, 81). PLGA MS uptake by human moDCs *in vitro* does not negatively influence biological properties, such as survival, cytokine secretion, antigen presentation or subsequent T cell stimulation (75, 82). Also, uptake of PLGA nanoparticles has been investigated using *in vitro* generated human and mouse DC population (83–85). Human moDCs, CD34+ stem cell-derived DCs and mouse BMDCs were able to engulf PLGA NP. Uptake of PLGA MS and NPs was prevented using cytochalasin B, which points to involvement of actin-polymerization during phagocytosis of PLGA particles (86, 87). In fact, it was shown that PLGA nanoparticles are partly internalized via fluid phase pinocytosis but also through clathrin-dependent receptor mediated endocytosis, while uptake of PLGA microparticles by DCs was achieved by non-specific phagocytosis (88).

### Present Challenges of PLGA Nanoparticle Mediated Cancer Vaccines

With respect to vaccine design, one must consider that nanoparticles with a size range of < 200 nm are able to directly enter the lymphatic vessel system from the interstitial space by diffusing through endothelial cell junctions. Additionally, NPs even can easily cross physiological barriers, such as the pulmonary tract, epithelial tight junctions or the blood-brain-barrier (BBB) without specific targeting. On the one hand, PLGA nanoparticles might facilitate stimulation of immune responses via direct delivery of antigens to lymph node (LN)-resident DCs and macrophages within hours after administration (82, 89). On the other hand, it has been established that premature antigen presentation may lead to induction of antigen tolerance. Furthermore, toxicity issues of unspecific uptake by other endocytic cells or non-specific distribution is still a problem of PLGA based nanoparticle-mediated vaccine delivery (90). In contrast, PLGA microspheres remain at the subcutaneous injection site in peripheral tissues and require active uptake by immature DCs resulting in proper activation of DCs and migration to skin-draining LNs where they efficiently present the processed antigens to naïve T cells. Additional toxicity concerns of nano-polymers have emerged, namely electrostatic interaction of positively charged nanoparticles with cell membranes, the recognition of hydrophobic NPs with cells of the reticuloendothelial system (RES) or aggregation of small cationic nanoparticles with serum proteins, potentially causing severe immunotoxicity by hemolysis or platelet aggregation (“nanotoxicology”) (90). To improve directed targeting and to minimize safety issues of undesired biodistribution *in vivo*—a problem we are not facing with the use of PLGA microspheres–nanoparticles need to be either surface-modified by hydrophilic moieties like the non-ionic polymer poly ethylene glycol (PEG) or need to be decorated with anchoring endocytosis molecules such as mannose, fucose, N-acteylglucosamine directed against DC-specific surface receptors (e.g. DC-SIGN, mannose receptor, DEC-205) or with DC-specific antibodies such as anti-CD11c (91, 92). The attachment of DC targeting moieties on PLGA NP surfaces has resulted in enhanced vaccine efficacy due to selective cellular binding, facilitated receptor-mediated endocytosis, and subsequent increased antigen cross-presentation to CD8+ T cells (93, 94). Despite enhancing homing mechanisms, preclinical and clinical data over the last decade have unveiled that targeting optimizations did not increase intratumor delivery of NP, which is below 1% of the injected nanoparticle dose (95, 96).

**Figure 3:**
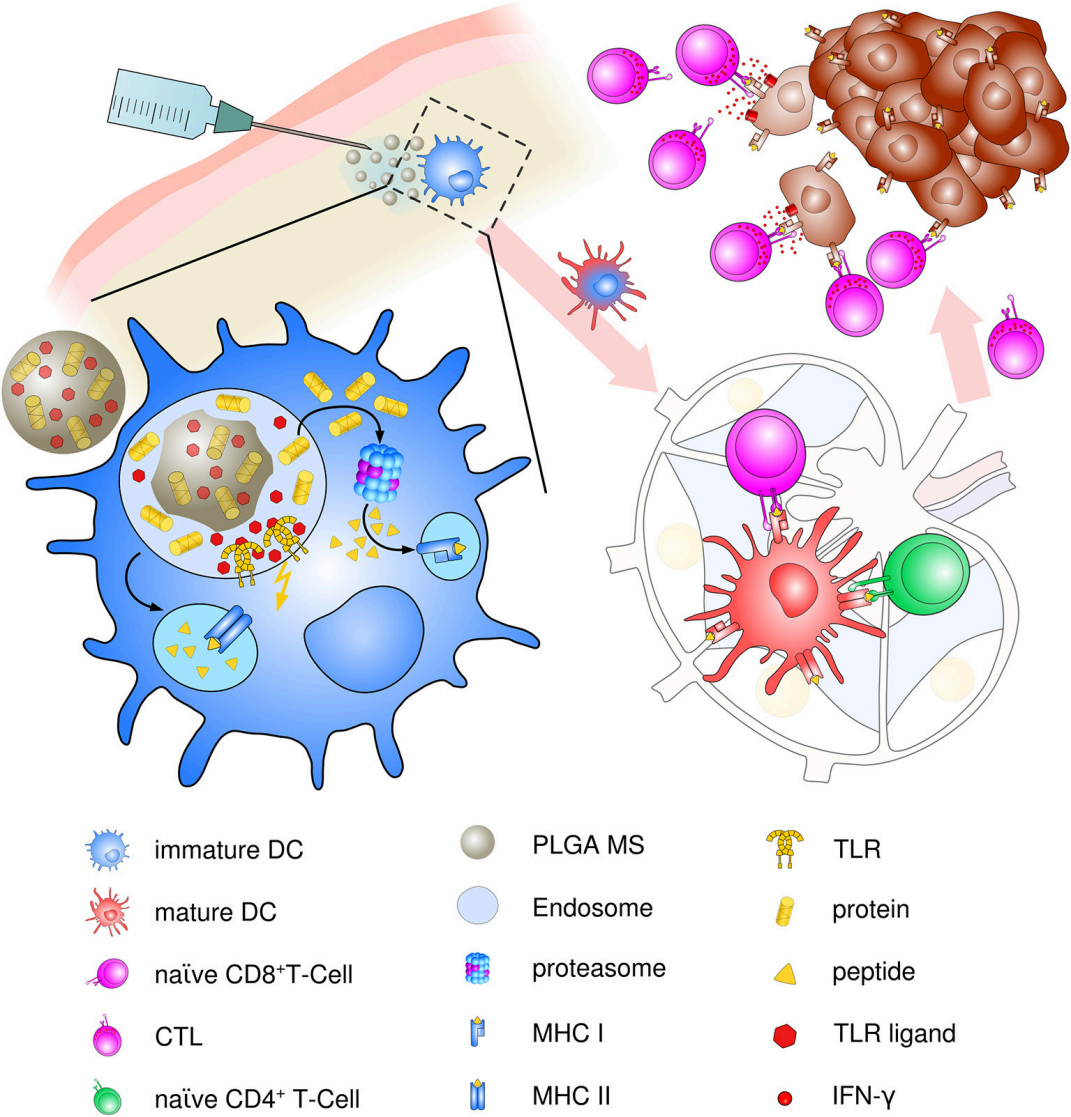
Schematic description of PLGA microsphere mediated anti-tumor response. After subcutaneous immunization, PLGA microspheres are efficiently taken up by immature, skin-resident APCs, mainly DCs. Co-delivery of antigens and TLR ligands leads to enhanced DC activation and maturation by upregulation of co-stimulatory surface maturation marker and MHC class molecules I and II during migration to lymph nodes. In the draining lymph node, encapsulated cancer antigens are processed and presented on either MHC class II to naive CD4+ T helper cells or via cross presentation to CD8+ T cells. Priming and activation of CD8+ T cells leads to differentiation and proliferation of tumor antigen-specific effector CTLs. Clonal expansion and CTL infiltration into the tumor environment results in recognition and eradication of target tumor cells mediated via IFN-γ release and enhanced Th1 polarized immune functions.

Though present particle-based cancer vaccine strategies have been built upon the hypothesis of preferential uptake of nanoparticles (smaller than 200 nm) and subsequent superiority at priming of cytotoxic responses over microparticles (>1 μm) (97), the optimum particle size for eliciting maximum immune responses has been a challenging topic ever since. Particle size is an important but not the only factor for dictating cellular uptake and intracellular trafficking. In contrast, the induction of specific and potent immune responses depends on a vast array of parameters including physico-chemical properties of PLGA, polymer composition, molecular weight and preparation methods, as well as routes of administration and nature and content of the encapsulated material. We suggest that PLGA microspheres exhibit an ideal adjuvant particle size inducing consistent and very effective immune responses *in vivo* that encourages ongoing use and future optimization of PLGA microsphere-based anti-cancer vaccines (see Figure 3).

## DC-mediated Antigen Presentation From PLGA Particles

Upon endocytic uptake of PLGA microspheres by iDCs, the particles are internalized into early endosomes. A combination of homogenous bulk polymer erosion and slow hydrolysis of microspheres leads to release of the micro-encapsulated antigens and molecules over a period of about 30–60 days, which elicits a low micro-environmental pH that further enhances PLGA hydrolysis (44). Inside the acidic endosomal compartment, lysosomal proteases, and peptidases cleave released antigens into peptides of 12–25 amino acids in length which normally enter classical endocytic pathway via MHC class II presentation for interaction with CD4+ T cells (98). Furthermore, release of the antigenic cargo, including TLR ligands with receptors located at the inner endosomal membrane, leads to endosomal acidification and maturation of the phagosome associated with TLR triggering (99). Reversion of the anionic particle surface charge (from negative to positive) in the acidic lysosomal compartment enables local interaction with endo-lysosomal membranes and facilitates escape from phagosomes into the cytoplasmic compartment. In fact, PLGA micro/nanoparticles rapidly escape the endo-lysosomal compartment within minutes (65, 76). Another possibility of endosomal escape has built upon the “proton-sponge mechanism.” The influx of chloride and hydronium ions during endosomal acidification causes osmotic pressure and leads to rupture of the endosomal membrane and subsequent release of its content (25, 100). Cytosolic release of the encapsulated proteins leads to antigen degradation into 8–11 aa long peptides by the proteasome before loading of these peptide fragments onto MHC class I molecules in the ER, a process known as “cross-presentation” (101). MHC class I—peptide complexes are subsequently transported to the cell surface to be presented to CD8+ T cells, thereby inducing the differentiation of CTLs. PLGA encapsulated antigens can be cross-presented by either endosomal escape (phagosome-to-cytosol pathway) or even simultaneously via the vacuolar pathway in the endocytic compartment. Compared to that, other particulate antigen formulations are exclusively relying on the TAP/proteasome-dependent pathway (102). Via exploiting distinct pathways of antigen presentation, PLGA-based particles increase the peptide pool that is presented on MHC class I and subsequently, the magnitude of the resulting CTL response. Furthermore, downregulation or loss of TAP activity is a major mechanism of tumor immune evasion (103). Thus, TAP deficiency in tumors won't necessarily hamper PLGA MS-mediated antigen cross-presentation by usage of the vacuolar pathway. Involvement of the cross-presentation pathway in processing of encapsulated protein and peptide antigens is further underlined by blockage of their presentation using proteasome inhibitors or brefeldin A (104). Cross-presentation is highly relevant for anti-tumor vaccines that rely on proper induction of tumor killing CTLs (25, 29, 38, 65, 79). Simultaneously, release of antigens into the cytosol may protect the antigenic content from further lysosomal degradation resulting in prolonged antigen presentation. Efficient presentation of PLGA MS delivered proteins and peptides onto MHC class I and II leads to development of a full-blown immune response, since activation of CD4+ T cells, particularly T helper 1 (Th1) cells, are central for activation and stimulation of antigen-specific CTLs through secretion of IFN-γ, IL-2, and IL-12. In addition to direct tumor cytolytic functions, IFN-γ secretion further recruits crucial mediators of the innate immune response, such as NK cells and macrophages thereby potentiating tumor cell killing or apoptotic tumor body clearance (105, 106). The only limitation of PLGA microparticles for use as anti-cancer vaccine is attributed to high initial burst due to dissolution of molecules that are adsorbed at the particle surface which may cause unintentional toxic side-effects (45). However, it has been demonstrated that the initial burst is of lower magnitude in larger (micro-)particles compared to smaller particles (46).

## Co-encapsulation of Antigen and Immunostimulatory Pattern Molecules

Encapsulation of antigen together with immunomodulatory molecules overcomes obstacles associated with present adjuvant containing vaccines. For instance, an ameliorated safety profile of adjuvants is accomplished by dose reduction, thus limiting undesired toxicities due to systemic administration of the immune potentiators at non-targeted tissues. Immunogenicity of the encapsulated antigen can further be improved or increased using immunostimulatory adjuvants through providing cellular, humoral, and/or mucosal immunity. Besides ensuring efficient antigen presentation due to proper DC activation and maturation, co-delivery of antigen and adjuvants in PLGA MS/NPs may further potentiate the induced immune response through secretion of NK cell recruiting and activating cytokines by the stimulated DC. Hence, activation of both CTL and NK cell mediated anti-tumor responses are able to eliminate MHC class I positive as well as negative tumors.

The choice of the adjuvant critically determines the outcome and spectrum of the elicited immune response. Thus, addition of adjuvants improves the induction of immune responses of poorly immunogenic tumor self-antigens and potentially supports reduction of the required antigen amount.

### Currently Used Adjuvant Agents in Vaccine Formulations

Delivery of both, the antigen and an appropriate DC maturation stimulus in physiological and temporal vicinity improves migratory capacity toward LNs and efficiently stimulates proper T cell responses. Indeed, T cell activation by single encapsulated antigens in the absence of costimulatory molecules or pro-inflammatory cytokines may induce Th2-associated unfavorable immune responses or may even result in tolerance induction against the antigen. The most common adjuvant which has been introduced for vaccination trials over 60 years ago is the water-in-oil emulsion incomplete Freund's adjuvants IFA (107), commonly used as Montanide™ ISA-51 in clinical trials of DC-based immunotherapy (108, 109). The adjuvant effect relies on formation of a local depot providing slow release and prolonged presentation of the antigen (110). Although, IFA is primarily known to induce Th2-biased responses and stimulating humoral responses of long-term IgG production, it can also stimulate CTL or Th1 immunity directed against the antigen that is emulsified in IFA (111). Due to emerging adverse effects such as local skin reactions, abscesses, inflammation or granulomas at the injection site, IFA is not allowed for routine immunotherapy (112). Aluminum salts (alum, and its derivate MF-59) were the first adjuvants approved by the FDA and EMA for clinical use in humans (113, 114) and are currently present in the composition of the majority of vaccines (115). Although generally well-tolerated, alum adjuvants skew immune responses toward humoral mediated and Th2-polarizing conditions and only poorly stimulate CTL responses (116), additional to critical safety concerns and poor therapeutic benefit (117). Apart from alum, there are only two other adjuvants clinically approved for human use, which are AS03 [used in the H5N1 vaccine Prepandrix®(118)] and AS04 (a combination of alum and TLR 4 ligand monophosphorly lipid A (MPL®) applied in hepatitis B virus (HBV, Fendrix®) and human papilloma virus (HPV, Cervarix®) vaccines) (119).

Toll-like receptor (TLR) ligands have been demonstrating a huge impact on cancer immunotherapy due to their capacities of DC activation and promotion of desired Th1 polarized immune responses. Several TLR ligands including oligonucleotides, single- or double-stranded RNA (ssRNA, dsRNA), flagellin or lipopeptides have already been investigated in clinical trials of a plethora of cancer types as reviewed in Temizoz et al. (120).

### Encapsulated TLR Ligands as DC Priming Adjuvants

TLR stimulation greatly enhances PLGA vaccine efficacy through powerful activation of DCs including the three signals required for proper T cell activation: increased expression of peptide-MHC complexes, upregulation of co-stimulatory molecules and cytokine secretion (121, 122). Furthermore, TLR triggering enhances cross-presentation to CD8+ T cells and stimulates a Th1-polarized immune response (123). Co-encapsulation of the antigen with either TLR7 or TLR9 ligands into PLGA MS stimulates DC maturation as well as cytokine secretion, and facilitates cross-presentation *in vitro* as shown by Heit et al. (124). Encapsulation of other so-called pathogen recognition receptor (PRR) agonists such as NOD (nucleotide-binding oligomerization domain-like receptor) ligands into either PLGA NP or MS have resulted in similar improvement of vaccine efficiency through enhanced maturation and pro-inflammatory cytokine secretion of human moDCs (125, 126). A detailed list of studies demonstrating improved cellular responses elicited by PLGA particulate systems via association of TLR ligands compared to the antigen alone or over soluble counterparts was extensively reviewed by Silva et al. (20).

We have incorporated at least two TLR ligands into our PLGA MS regimen, which were chosen due to their described Th1 inducing immunomodulation and stimulation of both humoral and cellular immunity (127, 128), namely CG-rich unmethylated Oligodeoxynucleotides (CpG ODN) and the RNA virus associated danger signal polyI:C (polyinosinic:polycitidylic acid) (29, 71, 129, 130). Their receptors, TLR9 and TLR3 respectively, are localized in the membrane of the endosomal compartments of most APCs where PLGA MS are internalized after endocytic uptake (131). Importantly, actual expression pattern of the respective TLRs has to be considered for particle vaccine design and the preferred targeting cell type. While TLR9 expression is limited to plasmacytoid DCs (pDCs), B cells and keratinocytes, TLR3 is expressed more broadly (132). Co-encapsulation of the model antigen ovalbumin together with CpG ODNs or polyI:C into PLGA microspheres efficiently elicited potent antigen-specific CTL responses and Th1 differentiation in comparison with soluble antigen after a single subcutaneous PLGA MS immunization *in vivo* (71, 130). Mice immunized with PLGA MS OVA/CpG generated a 2-fold increase in antigen-specific CD4+ and CD8+ T cell proliferation and IFN-γ production compared to a mixture of MS loaded separately with either the antigen or the adjuvant (PLGA MS OVA + PLGA MS CpG) (71). Pulsing of DCs with empty PLGA microspheres did not induce DC maturation *in vitro* (130), nor did vaccination of mice with empty PLGA MS elicit undesirable T cell responses (36, 71, 79, 133) thus confirming antigen-specificity of immune responses induced with PLGA microspheres. In stark contrast to that, pro-inflammatory adjuvant properties of PLGA microparticles (in comparison to PLGA nanoparticles) have been observed in macrophages (134). Several other TLR agonists have proven strong potential of enhancing the immunogenicity and efficacy of PLGA particle mediated cancer therapy in preclinical settings such as the TLR4 ligand monophospholipid A (MPLA), a chemically modified derivative of the *S. minnesota* derived endotoxin lipid A (135). Indeed, co-administration of TLR agonists in protein and peptide based cancer vaccines have entered clinical phase such as the TLR3 ligand poly ICLC (Hiltonol®) demonstrating tumor regression of advanced facial rhabdomyosarcoma (136), or the TLR7 agonist Imiquimod which has been approved for treatment of basal cell carcinoma due to its ability of CTL-mediated tumor regression by DC and NK cell recruitment (137).

### Enhancing PLGA Mediated Cancer Vaccines by Co-delivery of a Second TLR Ligand

Improvement of the PLGA MS system by adding a second TLR ligand, separately encapsulated has been shown to positively influence Th cell polarization to Th1—mediated immune response by targeted DCs (71, 129), suggesting that optimal DC activation depends on synergistic triggering of several TLR signaling pathways (138). Immunization of mice with PLGA MS OVA/CpG together with PLGA MS polyI:C resulted in greater number of KLRG1+ effector T cells (139) and increased cytotoxic effector functions of OVA-specific CD8+ CTLs, as demonstrated by IFN-γ production, oncolytic granzyme B and perforin secretion and increased CD107α expression (71). Several other groups have similarly demonstrated that concomitant delivery of antigen and adjuvant in the same endo-lysosomal compartment is required for proper activation of DCs and superior CTL induction *in vivo* (124). In any case, cross-presentation of the internalized antigen was enhanced with simultaneous co-encapsulation of either TLR3 or TLR9 ligands and the antigen (140, 141). The enhanced vaccine efficiency manifests in prolonged presentation of antigen derived epitopes and superior anti-tumor responses in mice (71, 124, 142). For example, co-delivery of PLGA NPs-OVA together with the TLR4 ligand MPLA (143) or the melanoma antigen TRP2 with another TLR4 ligand (7-acyl lipid A) (144) generated improved antigen-specific responses.

Surprisingly, other studies have come to the opposite conclusion, namely that co-administration of antigen and TLR ligand in different PLGA particles [PLGA NP OVA + PLGA NP (MPLA + R837)] yields better results compared to co-delivery of antigen and adjuvant in the same nanoparticle (PLGA NP OVA/MPLA/R837). Mentionable, these studies only focused on the humoral response and IgG1 and IgG2a production and have not analyzed cellular immunity. Of note, TLR7 (the receptor for R837) is not expressed in the cross-presenting CD8α+ splenic DC subset (145) which may cause inferior responsiveness toward imidazoquinolines. Moreover, the discrepancy between co-encapsulation and co-administration strategies probably depends on the particulate nature, the encapsulated antigen, the route of administration and the choice of adjuvant. Another possibility to enhance immunogenicity of PLGA MS mediated vaccine delivery system is co-encapsulation of multiple specific CTL epitopes. It was already demonstrated that administration of two OVA-derived epitopes into one PLGA microsphere elicited substantial IFN-γ secretion *in vivo* (146).

## Anti-tumor Responses to Immunotherapy With PLGA Particles

Co-delivery of antigen and adjuvant to DCs is required for PLGA MS-mediated anti-tumor immunotherapy. Reduction of tumor growth in various syngeneic tumor models in mice was better compared to the same antigen emulsion in IFA. Both, protective as well as therapeutic treatment with PLGA MS OVA/CpG + PLGA MS polyI:C elicited potent anti-tumor activity in subcutaneous tumor models as well as in lung metastasis models using EG-7 thymoma or the aggressive MO-5 melanoma tumor cells in mice (129). Remarkably, even a single administration of co-encapsulated OVA/CpG microspheres completely protected mice from tumor growth (129). Increased anti-tumor activity using PLGA associated nanoparticulate vaccines was shown by others as well. PLGA NP OVA/polyI:C or PLGA NP OVA/CpG exerted potent anti-tumor activity against subcutaneously implanted EG-7 tumor cells (147). Noticeably, Silva et al. demonstrated decreased growth of B16F10 melanoma in both therapeutic and prophylactic settings using MHC class I or II restricted melanoma peptides Melan-A and gp100 encapsulated into PLGA NP together with either one or both of the TLR3 and TLR9 ligands polyI:C and CpG (148). This study offered several distinct conclusions besides confirmation of the fact that co-encapsulation of antigens and adjuvants in PLGA particles improves antigen-specific anti-oncogenic immunity. First, the study shows synergistic effects of enhanced anti-tumor activity by co-encapsulation of the two immunopotentiators CpG and poly(I:C) into one particle. Second, mice were slightly more protected from tumor growth after immunization with nanoparticles containing two MHC class I-restricted melanoma epitopes simultaneously. Furthermore, the authors propose that co-administration of PLGA NPs with either an MHC class I or an additional MHC class II restricted epitope along with both TLR ligands induced almost complete blockage of tumor growth, suggesting the important activation of both CD8+ and CD4+ T cell responses for efficacy of anti-tumor immunity. IFN-γ secreting CD4+ T cells facilitate the differentiation of tumor antigen-specific CTLs and promote the recruitment of cells from the immune system participating in tumor cell containment (149). Further, tumor specific CD4+ T cells regulate the survival of CD8+ memory T cells (150). Combined TLR ligation on DCs triggering both MyD88 dependent and independent TLR mediated signaling pathways in parallel has already been demonstrated to promote broader activation of DCs. The marked increase in pro-inflammatory cytokine production and expression of co-stimulatory molecules resulted in enhanced T cell responses *in vivo* or even insensitivity to the immunosuppressive activity of Tregs at tumor sites (151–153). Additionally, tumor-induced immunosuppression of DCs is one of the main causes for ineffective anti-tumor responses (154). Thus, co-delivery of tumor antigens together with TLR ligands in PLGA MS not only targets the antigen to DCs, but might also rescue impaired DC function from tumor induced immunosuppression (155, 156).

### Alternatives to TLR Ligands as Immunomodulatory Compounds

In addition to TLR ligands, it is possible to include lipid antigens (e.g. the extremely potent glycosphingplipid α-galactosyl-ceramide, α-GalCer), which activate natural killer T (NKT) cells by binding to the non-classical MHC CD1 molecules. This unique subset of the T cell lineage acts as a potent adjuvant in immune responses against cancer by downstream activation of both innate and adaptive immune responses (157). Although not directly killing tumor cells, NKT cells simulate the cross-priming of tumor antigens by DCs through rapid secretion of large amounts of IFN-γ, IL-12, and IP10 (IFN-γ inducible protein 10) and are able to induce further recruitment of NK cells, macrophages, DCs, CD4+ and CD8+ T cells to tumor sites (157). A combination of α-GalCer and the TLR4 agonist MPLA into PLGA microspheres markedly increased cellular immune responses (158). Moreover, co-encapsulation of the invariant NKT cell agonist, together with the TLR 7/8 agonist R848 (Resiquimod) and polyI:C into PLGA nanoparticles enhanced CD4+ and CD8+ mediated anti-tumor responses mainly dependent on DC condition via NKT cells (159). Interestingly, a non-glycosidic derivate of α-GalCer, threitolceramide (ThrCer) has already proven clinically effectiveness in human and mice (160).

### Tumor Lysate as Antigen Source for Particulate PLGA Mediated Cancer Immunotherapy

As outlined above, endogenous and exogenous antigen supply in DC-mediated cancer immunotherapy has faced major limitations such as peptide degradation, rapid turnover of peptide/MHC complexes or dissociation of peptide from MHC during DC preparation/injection (161). This was likely attributed to the fact that only a limited number of peptides with few if any T helper peptides were used (162). Additionally, immunotherapy of solid malignancies is often hampered by low numbers of tumor-specific T cells due to inefficient antigen delivery of DC-based immunotherapy. Moreover, re-administered DCs displayed poor migratory capacity, thus limiting the amount of antigen presented to T lymphocytes in local dLN (163). The use of whole tumor lysates (TL) bypasses the limited potency of single antigen delivery thus broadening the repertoire of defined TAAs and neoantigens and thereby enhancing the probability of generating polyvalent, tumor-associated and antigen-specific CTL responses. Simultaneous stimulation of both CD8+ restricted CTL responses and CD4+ T helper cells is potentially complex enough to overcome the ability of tumors to down-regulate targeted antigens. PLGA MS co-encapsulating TRAMP-prostate derived tumor lysate and TLR ligands showed promising *ex vivo* cytotoxic T lymphocyte responses and achieved elimination of large tumor masses *in vivo* in TRAMP mice, a transgenic mouse model for prostate cancer (133). The anti-tumor efficacy of tumor lysate co-encapsulated with CpG ODNs in PLGA MS was also shown by Goforth et al. in a mouse model for melanoma (164). As well, a prime boost regimen of microspheres containing lysates of mammary gland tumor cells followed by a booster vaccination of bulk tumor lysate together with TLR ligands in liposome formulation was able to ameliorate tumor growth in a murine breast cancer model (165). Noticeably, patient-derived DCs loaded with PLGA NPs encapsulating lysed tumor tissue from patients with advanced head and neck squamous cell carcinoma (HNSSC) could efficiently induce IFN-γ production and could significantly reduce IL-10 secretion in autologous CD8+ T cells (166). Similar findings were made by Hanlon et al. demonstrating increased production of pro-inflammatory cytokines in healthy donor DCs that were pulsed with PLGA nanoparticles encapsulating tumor lysate of an ovarian cancer cell line (167). Malignant cells have developed prodigiously smart mechanisms to co-opt immune cells for tumor progression thereby creating an immunosuppressive microenvironment. Cancer cells are able to attract immunosuppressive cell types such as regulatory T cells (Tregs) and myeloid derived suppressor cells (MDSCs) and are known to drive TAM (tumor-associated macrophage) differentiation to the pro-tumorigenic M2 phenotype. PLGA MS mediated cancer therapy might be an ideal strategy to revert these immunosuppressive mechanisms by inducing factors that are essential for cytotoxicity against cancer cells such as intra-tumoral activated CD8+ T cell response and IFN-γ production as well as recruitment of NK cells. Moreover, upregulation and overexpression of immune checkpoints CTLA-4 (cytotoxic T lymphocyte associated antigen 4) or PD-L1 (programmed cell death protein ligand 1) on cancer cells induces T cell anergy and maintains Treg induced immunosuppression. Thus, it might also be interesting to combine PLGA microsphere-based immunotherapy with immune checkpoint inhibitors to restore T cell anti-tumor effector function.

Apart from generation of anti-tumor responses, we additionally could demonstrate the preeminence of PLGA MS in infectious diseases. PLGA MS encapsulated Influenza virus matrix M1 peptide together with CpG induced potent anti-viral CTL responses and protected against Influenza A infection (168).

In relation to a potential use of PLGA MS in clinical application, sterilization of PLGA MS by γ-irradiation did not negatively affect T cell responses (133). The biggest advantage of spray-dried PLGA microspheres is the high reproducibility of the low-cost MS production meeting GMP requirements of efficacy, safety and stability of pharmaceuticals.

## Future Perspectives

PLGA microspheres have demonstrated great proficiency for potential use in cancer immunotherapy (see Table 1). They have overcome the major challenges of drug delivery systems, such as protection of encapsulated material from rapid degradation and clearance. PLGA MS exhibit ideal properties for facilitated and untargeted uptake of mainly DCs after subcutaneous injection. Concomitant delivery of antigens and adjuvants to the same APC leads to efficient DC activation and increased stimulation of CD4+ T cells as wells as of CD8+ T cells via cross-presentation by coordinate and synergistic pathways. PLGA MS mediated drug delivery allows particularly low doses of antigens and adjuvants–still inducing strong CTL responses but minimizing potential side-effects of unspecific activation of systemic immune responses. Reducing the doses of antigen or immunostimulants is generally desired regarding potential clinical application or approval by international regulatory agencies. Sustained and prolonged antigen release induces superior immune responses and CD8+ T cell memory while simultaneously avoiding the risk of tolerance induction. The depot effect created at the injection site substitutes the need for conventional booster injections to maintain immune responses. Co-encapsulation of antigens together with toll-like receptor ligands yields potent and long-lasting CTL and T helper cell responses *in vivo* leading to protective and therapeutic anti-tumor activity in several tumor mouse models.

**Table 1:** Main advantages of PLGA microspheres as a DC-mediated particulate vaccine delivery system for cancer immunotherapy.

	**Advantage**	**Rationale**
General issues PLGA	Biodegradability and biocompatibility Approval for parenteral use by regulatory authorities Versatile physico-chemical properties (e.g. MW, L:G ratio) Encapsulated material is protected from premature degradation Possibility of polymer surface modification	Complete degradation into toxicologically safe metabolic products Improved safety; commercially available as cGMP product Adjustable drug release profile; tailoring of elicited responses Preserving therapeutic activity e.g. addition of targeting moieties
Spray Drying method	Long-term storage in powder form Few processing parameters, short manufacturing time Low amount of residual organic solvent; no addition of stabilizers/emulsifiers needed High reproducibility between spray-dried batchesd High drug loading and encapsulation efficiency	Physical and chemical stability at 4°C without loss of biological activity Adjustable particle size and shape; ease of industrial scalability No adverse effects due to solvent impurities Standardized and compendious protocolsd Reduced antigen/adjuvant doses minimize side effects or systemic immune activation by soluble immunomodulators
Boosting the immune response	Sustained and controlled release for extended time-period (≥30 days); depot effect at the injection site Particle sizes of 0.5–5 μm via spray-drying Enhanced & prolonged antigen (cross-) presentation on MHC class I and II Concomitant delivery of antigens and adjuvant in the same PLGA microsphere Induction of strong effector CTL responses Single shot cancer vaccine Microencapsulation of whole tumor lysate	Avoids risk of antigen tolerance; substitutes need for booster injectionsd Passive but facilitated internalization by APCs, particularly DCs Activation of adaptive and humoral immunity; cross-presentation of tumor antigens; stimulation of CTL and Th1 responses Enhanced, direct endolysosomal delivery in target cells, synergistic interaction of DC activation and T cell stimulation Increased immunogenicity of peptides or tumor antigens Reduction or complete protection of tumor growth; induction of long-term memory Enhanced anti-tumor activity; possibility of personalized vaccination

Despite the mentioned advantages of PLGA particles, particulate cancer vaccines are not available for clinical application at present. By far, most *in vitro* and preclinical mouse studies have been performed with model antigens and model tumors. It is important to switch to clinically relevant antigens and autochthonous, transgenic or carcinogen-induced tumor models for more realistic efficacy assessments in the future. Moreover, the production of GMP-grade PLGA MS needs to be established and refined to get approval for clinical studies. Translation form bench-side into the clinic has always been challenging due to various aspects including characterization of all materials used, availability of cGMP products, the presence of residual organic solvent impurities, difficulties in controlling encapsulated drug release including high initial burst and incomplete release, variability in particle size or morphology between different batches and safety issues including effectiveness and ease of administration in human cancer patients. Increasing the implementation of process analytical technologies (PAT) will control manufacturing and development of PLGA particles to ensure reproducible, effective and safe vaccines and clinical transition. The spray drying process would overcome limits of applicability in larger clinical settings, since the production of PLGA MS is easy to scale-up, cost-effective and amenable to sterile manufacturing. Unlike vaccines for infectious diseases, cancer vaccines might need to be tailored for individual patients due to diverse gene mutations in cancer cells creating neo-antigens. Hence, the development of custom-designed whole tumor lysate encapsulated into personalized PLGA MS might introduce a very promising, rapid and potent cancer treatment approach. Tumor lysates provide a pool of tumor-associated antigens to trigger suitable CD8+ and CD4+ T cell mediated anti-tumor responses that overcome the infirmities of single peptide vaccinations. Currently we are investigating PLGA MS mediated immune responses of used immunostimulatory molecules in Vaccigrade™, GMP certified and endotoxin-free formulations as well as other adjuvant candidates.

In summary, concomitant delivery of antigens and immunomodulators in PLGA microparticles reveals a potent DC—centered therapeutic approach for inducing strong anti-tumor immunity in various cancer settings which might pave the way for PLGA microspheres to become a key member of current cancer vaccines.

## Author Contributions

JK wrote the manuscript. DH prepared the figures. MG supervised associated projects and corrected and refined the manuscript.

### Conflict of Interest Statement

The authors declare that the research was conducted in the absence of any commercial or financial relationships that could be construed as a potential conflict of interest.
